# The influence of regular monitoring and exercise on employee health: A study at Cicendo Eye Hospital measuring differences between clinical and non-clinical staff

**DOI:** 10.1016/j.puhip.2026.100762

**Published:** 2026-03-20

**Authors:** Antonia Kartika, Aaron Tigor Sihombing, Mayasari Wahyu, Elfa Ali Idrus, Hartono, Nana

**Affiliations:** a National Eye Center, Cicendo Eye Hospital, Bandung, Indonesia; b Department of Ophthalmology, Universitas Padjajaran, Bandung, Indonesia; c Department of Surgery, Division of Urology, Universitas Padjajaran, Bandung, Indonesia; d Hasan Sadikin General Hospital, Bandung, Indonesia

**Keywords:** Occupational health, Workplace wellness program, Health screening, Exercise intervention, Indonesia

## Abstract

**Objectives:**

The Indonesian Ministry of Health introduced mandatory health screening and exercise programs for hospital staff to improve well-being. This study compared health outcomes between clinical and non-clinical personnel participating in the program.

**Study design:**

Retrospective cohort study.

**Methods:**

This study used data from Cicendo Eye Hospital's mandatory quarterly health screenings and exercise program records. Our analysis included full-time employees from both clinical and non-clinical roles who completed three screening sessions. Biometric data, including body mass index, waist circumference, blood pressure, random blood glucose, and cholesterol levels, were collected at baseline and at 3- and 6-month follow-ups. Repeated-measures analyses were used to evaluate temporal changes.

**Results:**

Of the 416-hospital staff, 362 (87%) met the inclusion criteria and were included in the final analysis. After a six-month follow-up, there was a significant reduction in BMI, waist circumference, and cholesterol levels across the study population. No significant changes were found in blood pressure. However, the study revealed a significant increase in the proportion of individuals with abnormal random blood sugar levels among the nurse and non-physician/non-nurse groups.

**Conclusion:**

During regular health monitoring and exercise programs, statistically significant temporal reductions in body weight, BMI, waist circumference, and cholesterol levels were observed during the 6-month follow-up period. among clinical and non-clinical hospital staff. However, the lack of change in blood pressure and the notable increase in abnormal blood sugar in certain professional groups, like nurses, underscore the need for tailored, group-specific wellness programs to effectively address the unique health risks of different personnel.

## 1 Introduction

Beginning in January 2025, the Indonesian Ministry of Health implemented a mandatory quarterly health screening program for all clinical and non-clinical staff at Ministry of Health hospitals throughout Indonesia [1]. The initiative aims to proactively shift health culture and screen for diseases to enhance staff productivity and reduce healthcare costs. Four key indicators were chosen for this initiative, reflecting common disease burdens in the Indonesian population, which are blood pressure, blood glucose level, lipid profile, waist circumference, and body mass index (BMI) [2].

Regular health checkups have been shown to positively influence both health behaviors and labor productivity, with individuals who receive checkups exhibiting healthier habits and greater productivity. According to a study from Kang and Kawamura in Japan, individuals who received health checkups demonstrated a positive association with healthier behaviors and higher labor productivity [3]. Specifically, these individuals worked longer hours and exhibited healthier habits than those who did not receive health checkups. This suggests that health checkups may lead to dual benefits, improving both personal health outcomes and labor market productivity.

Effective clinical preventive medicine programs can significantly reduce employee illness risk factors, leading to decreased absenteeism and higher productivity [4]. In the healthcare industry, where preventable illnesses and poor health-related performance can severely impact operations, implementing preventive healthcare is especially promising. This is particularly true in service sectors where individual employee performance is crucial to success [5].

Healthcare professionals, including doctors, nurses, and administrators, face a unique set of occupational hazards that directly impact their health. The World Health Organization (WHO) has identified several of these risks, such as long work hours, high-stress environments, and elevated risks of physical injury and mental health disorders [6]. The intense pressure and irregular schedules common in this field often lead to poor lifestyle choices, including unhealthy diets, reduced physical activity, and inadequate sleep. These behavioral changes can result in negative physiological outcomes, such as an increased BMI, a larger waist circumference, and unfavourable changes in lipid and blood sugar levels. Therefore, a workplace health program in a hospital is a necessary intervention to counteract the physiological effects of the demanding work environment, not just an added benefit.

The quarterly health screening program at Cicendo Eye Hospital, an Indonesian Ministry of Health hospital, is particularly relevant because it targets a known issue within a highly pertinent population: hospital staff. This initiative employs interventions based on established best practices. By evaluating its effects on specific, measurable biometric markers, the study seeks to create an evidence-based framework for similar programs globally. However, there is a lack of research examining the differential impact of such programs on clinical versus non-clinical hospital staff, who face distinct work demands and stressors. Therefore, the primary objective of this study is to measure the differences in health outcomes between clinical and non-clinical staff following their participation in the mandatory quarterly health screening program and a supplementary exercise program, thereby providing a more nuanced understanding of how these interventions affect distinct groups within a hospital setting.

## 2 Methods

### 2.1 Study design

This study is a retrospective cohort analysis conducted at Cicendo Eye Hospital in Bandung, Indonesia. The hospital is a national eye center and a government owned hospital under the Ministry of Health.

### 2.2 Study population

The study population consists of full-time employees of Cicendo Eye Hospital who participated in the comprehensive in-house healthy lifestyle program from January 2025 to July 2025. The program included health education sessions, weekly morning walks, and quarterly biometric monitoring. The study cohort is stratified into three professional roles: physician, nurse, and non-physician/non-nurse. Only individuals who participated in all three measurement sessions were included in the study analysis.

### 2.3 Intervention

The hospital-wide healthy lifestyle program was implemented as an institutional policy and consisted of three integrated components:

1. Health education: In-house educational sessions delivered after baseline data collection in January 2025, covering nutrition, physical activity, and general lifestyle modification.
2. Exercise activities: Monthly group-based morning walks combined with dance-based aerobic exercise, conducted on hospital grounds on the third Friday of each month.
3. Biometric monitoring: Mandatory quarterly on-site health screenings measuring body mass index (BMI), waist circumference, blood pressure, random blood glucose, and total cholesterol.

Participation in biometric monitoring was mandatory for all full-time staff, while individual adherence to education and exercise components could not be quantitatively assessed. Therefore, exposure intensity to specific intervention components was not measured.

### 2.4 Data collection

Data were collected retrospectively from the hospital's electronic health records and the Human Resources Department database. Biometric data, including body mass index, waist circumference, blood pressure, random blood glucose, and cholesterol levels, were collected at three predefined time points: baseline (January 2025), 3 months (April 2025), and 6 months (July 2025). All analyses were conducted using these standardized time points to ensure consistency in longitudinal assessment.

Demographic information, including age, gender, and professional role, was also collected.

### 2.5 Outcome measures

The primary outcome measures are the changes in the following biometric markers from baseline to 6 months:

• BMI
• Waist circumference
• Blood pressure
• Random blood glucose
• Total cholesterol

### 2.6 Statistical analysis

Descriptive statistics were used to summarize demographic and baseline biometric characteristics across occupational groups. Continuous variables are presented as means ± standard deviations, while categorical variables are presented as frequencies and percentages.

Changes in biometric parameters over time were analyzed using within-subject repeated-measures analyses. Prior to longitudinal analysis, data distribution was assessed using the Shapiro–Wilk test and visual inspection of histograms and Q–Q plots. Repeated-measures ANOVA was applied for normally distributed variables, while the Friedman test was used as a non-parametric alternative when normality assumptions were not met. Analyses were performed to assess temporal changes within the cohort and to explore differences in trends across occupational groups. No external or internal comparator group was used. Statistical significance was defined as *p* < 0.05. All analyses were conducted using IBM SPSS Statistics version 29.0.

## 3 Results

From an initial pool of 416 hospital staff, 362 individuals (87%) completed all three measurement sessions. Table 1 outlines their professional and baseline characteristics.

**Table 1:** Professional and baseline characteristics.

Parameters	Physician		Nurse		Non-Physician/Non-Nurse Staff	
	Male	Female	Male	Female	Male	Female
n (%)	10 (30.3)	23 (69.7)	38 (26.2)	107 (73.8)	86 (45.3)	104 (54.7)
Age	47.11 ± 9.33	47.70 ± 8.47	42.00 ± 7.20	44.20 ± 8.51	43.92 ± 7.87	42.40 ± 8.64
	47.53 ± 8.57		43.62 ± 8.22		43.08 ± 8.31	
Body Weight	78.65 ± 8.20	63.63 ± 10.97	74.66 ± 14.23	62.93 ± 10.71	72.07 ± 13.02	63.34 ± 12.69
Body Height	171.45 ± 7.54	156.61 ± 6.37	167.05 ± 7.59	155.72 ± 4.51	166.65 ± 6.38	153.50 ± 5.48
BMI Category						
Underweight	0 (0.0)	0 (0.0)	0 (0.0)	0 (0.0)	2 (2.3)	2 (1.9)
Normal	1 (10.0)	5 (21.7)	5 (13.2)	28 (26.4)	22 (25.6)	21 (20.2)
Overweight	1 (10.0)	4 (17.4)	6 (15.8)	19 (17.9)	15 (17.4)	15 (14.4)
Obese	8 (80.0)	14 (60.9)	27 (71.1)	59 (55.7)	47 (54.7)	66 (63.5)
Waist Circumference	95.10 ± 5.45	83.24 ± 7.05	94.08 ± 10.97	83.62 ± 8.91	93.35 ± 10.14	85.12 ± 8.92
Blood Pressure						
Normal	2 (20.0)	10 (43.5)	13 (34.2)	51 (47.7)	28 (32.9)	50 (48.1)
Pre-Hypertension	3 (30.0)	10 (43.5)	14 (36.8)	29 (27.1)	31 (36.5)	29 (27.9)
Hypertension	5 (50.0)	3 (13.0)	11 (28.9)	27 (25.2)	26 (30.6)	25 (24.0)
Random Blood Glucose						
<140 mg/dL	10 (100.0)	23 (100.0)	34 (91.9)	104 (98.1)	80 (93.0)	101 (97.1)
140-199 mg/dL	0 (0.0)	0 (0.0)	3 (8.1)	1 (0.9)	2 (2.3)	1 (1.0)
≥200 mg/dL	0 (0.0)	0 (0.0)	0 (0.0)	1 (0.9)	4 (4.7)	2 (1.9)
Cholesterol Level						
Normal	6 (60.0)	11 (47.8)	14 (36.8)	53 (49.5)	31 (36.0)	57 (54.8)
High (≥200 mg/dL)	4 (40.0)	12 (52.2)	24 (63.2)	54 (50.5)	55 (64.0)	47 (45.2)
Mean ± SD	195.89 ± 39.90	200.47 ± 41.36	207.16 ± 39.21	198.78 ± 35.89	210.33 ± 38.83	195.42 ± 42.90

The changes in body weight, BMI, waist circumference, blood pressure, blood glucose, and cholesterol levels over the 6-month post-intervention period are summarized in Table 2. Table 2 demonstrates a statistically significant reduction in body weight, body mass index (BMI), waist circumference, and cholesterol; however, no significant change was observed in blood pressure. Conversely, a significant increase in random blood glucose levels was noted. Analysis was then performed based on occupational category.

**Table 2:** changes in body weight, BMI, waist circumference, blood pressure, blood glucose, and cholesterol levels.

Parameters	Baseline	3 Month	6 Month	p-value
Body Weight	66.87 ± 13.12	65.88 ± 12.88	66.23 ± 12.91	<0.001
BMI Category				
Underweight	4 (1.1)	7 (1.9)	7 (1.9)	<0.001
Normal	80 (22.0)	87 (24.0)	79 (21.8)	
Overweight	59 (16.3)	63 (17.7)	70 (19.3)	
Obese	220 (60.6)	206 (56.7)	207 (57.0)	
Waist Circumference	87.69 ± 10.30	86.53 ± 10.44	86.13 ± 10.23	<0.001
Blood Pressure				
Normal	153 (42.1)	146 (40.2)	154 (42.4)	0.062
Pre-Hypertension	114 (31.4)	150 (41.3)	152 (41.9)	
Hypertension	96 (26.4)	67 (18.5)	57 (15.7)	
Random Blood Glucose				
<140 mg/dL	349 (96.1)	331 (91.2)	328 (90.4)	<0.001
140-199 mg/dL	7 (1.9)	21 (5.8)	28 (7.7)	
≥200 mg/dL	7 (1.9)	11 (3.0)	7 (1.9)	
Cholesterol Level				
Normal	168 (46.3)	187 (51.5)	250 (68.9)	<0.001
High (≥200 mg/dL)	195 (53.7)	176 (48.5)	113 (31.1)	
Mean ± SD	201.43 ± 39.61	198.10 ± 38.75	178.15 ± 42.33	<0.001

Statistical Test: Friedman Test, except for Mean ± SD, ANOVA of Repeated Measure was used.

**Fig. 1:**
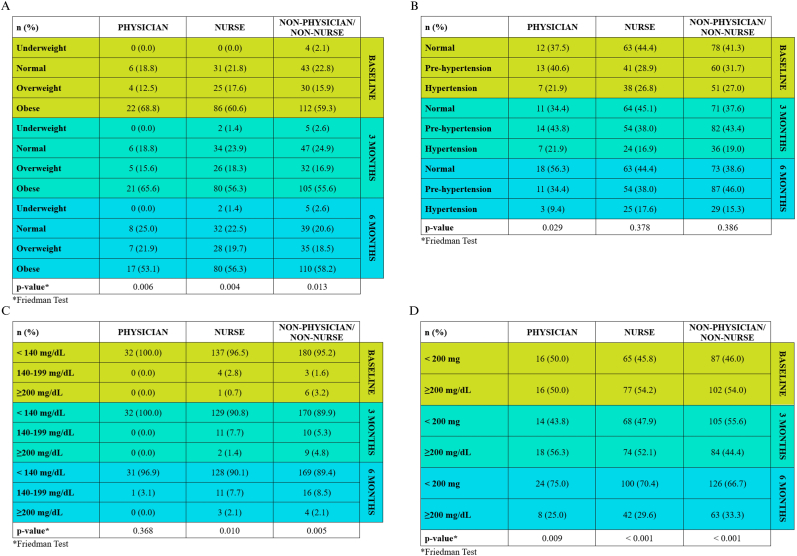
Parameter changes across time points stratified by Occupation. (A) BMI, (B) blood pressure, (C) random blood glucose, (D) cholesterol level.

At the 6-month follow-up, Fig. 1A shows that the physician cohort demonstrated a statistically significant increase in the normal and overweight IMT categories, alongside a significant decrease in the obese category. A similar significant trend was observed across other occupational groups, which also showed an increase in the underweight category.

Analysis of the data in Table 3 revealed a progressive reduction in waist circumference among the nurse and non-physician/non-nurse groups, with a statistically significant mean reduction of 1 to 2 cm at 6 months. Conversely, the physician group exhibited no statistically significant trend in waist circumference.

**Table 3:** Waist circumference changes based on occupation.

Waist Circumference		Baseline	3 Months	6 Months	p-value
Physician	Mean ± SD	86.64 ± 8.61	86.22 ± 8.65	85.75 ± 8.70	0.260
	Median (Range)	83.5 (72.0-104.0)	84.0 (70.0-104.0)	85.0 (68.0-102.0)	
Nurse	Mean ± SD	86.48 ± 10.55	85.78 ± 10.29	84.99 ± 10.10	<0.001
	Median (Range)	85.0 (65.0-118.0)	85.0 (65.0-116.0)	84.0 (65.0-115.0)	
Non-Physician/Non-Nurse	Mean ± SD	88.79 ± 10.32	87.19 ± 10.88	86.99 ± 10.60	<0.001
	Median (Range)	88.0 (66.0-124.0)	87.0 (51.0-122.0)	86.0 (64.0-121.0)	

Statistical Test: Friedman Test.

As shown in Fig. 1B, there were no statistically significant changes from baseline in any study group at the 6-month follow-up. Fig. 1C shows no statistically significant change in the random blood glucose category for the physician group. In contrast, A statistically significant increase in the proportion of individuals classified with abnormal random blood glucose levels was observed in the nurse and non-physician/non-nurse groups over the 6-month follow-up period. Longitudinal data in Fig. 1D show a significant progressive reduction in the proportion of individuals with abnormal cholesterol levels within each occupational group.

## 4 Discussion

This study is a retrospective cohort analysis evaluating within-group temporal changes in biometric health indicators among hospital staff participating in a mandatory institutional health screening and exercise program. The analysis, which stratified participants by their professional role, found that this short-term, in-house wellness program significantly improved several key health metrics. However, the effects varied among different occupational groups. This study should be interpreted in light of its observational design. As no comparator group was included, the findings reflect within-group temporal changes rather than causal effects of the health screening or exercise program.

At baseline, as shown in Table 1, the prevalence of obesity among all employee groups at Cicendo Eye Hospital was higher than both the national and West Java provincial averages. For context, the national prevalence of obesity among adults over 18 years old in Indonesia was 14.4%, while in West Java province it was 14.1% [7]. In contrast, the prevalence of obesity at Cicendo Eye Hospital was markedly higher: 68.8% for physicians, 60.6% for nurses, and 59.3% for non-physician/non-nurse staff.

The health screening program implemented for Cicendo Eye Hospital staff yielded significant positive outcomes, as evidenced by a comprehensive cohort analysis. Statistically significant reductions in anthropometric measures (body weight, BMI, waist circumference) and cholesterol levels were observed at the six-month mark (Table 2). This correlation aligns with established research indicating that structured health interventions facilitate better health awareness and management. Prior studies have documented that regular checkups are associated with self-reported good health and a reduced propensity for self-treatment due to improved health literacy [8,9]. However, the magnitude of change in BMI categories warrants cautious interpretation. Although the proportion of individuals classified as obese decreased from 220 participants (60.6%) at baseline to 207 participants (57.0%) at six months, this represents a modest absolute reduction of 13 individuals over the study period. While statistically significant, the clinical impact of this short-term shift may be limited. These findings suggest early directional improvement rather than substantial metabolic transformation, and longer follow-up would be necessary to determine whether these changes translate into meaningful reductions in cardiometabolic risk.

Interestingly, while there was a significant reduction in high cholesterol levels across all hospital staff (Fig. 1D), our data revealed a significant increase in random blood glucose levels among nurses and the non-physician/non-nurse group (Fig. 1C). The observed increase in abnormal random blood glucose levels among nurses and non-physician/non-nurse staff should be interpreted with caution. Random blood glucose measurements are subject to short-term variability and may be influenced by recent dietary intake, stress, or work schedules. In addition, this study did not collect data on dietary patterns, medication use (including glucose-altering drugs), or fasting status. It is also possible that this effect is linked to statin use, as several studies have shown that statins can increase the risk of elevated blood sugar in susceptible individuals [[10], [11], [12], [13]]. Consequently, this finding should be regarded as exploratory and descriptive, rather than indicative of a causal effect of the health screening or exercise program. Nevertheless, it may serve as a signal for future studies to incorporate more precise glycemic measures, such as fasting plasma glucose or HbA1c, and to account for relevant behavioral and pharmacological factors.

Although a trend toward reduced hypertension prevalence was observed across the entire hospital staff, the proportion of individuals in the pre-hypertension category increased among nurses and the non-physician/non-nurse group (Fig. 1B). The lack of a significant change in blood pressure within these two groups highlights the need for more targeted interventions. Despite the fact that the six-month follow-up prevalence of hypertension in these groups (17.6% and 15.3%, respectively) was lower than the West Java provincial average (39.6%), the large number of individuals with pre-hypertension and the absence of a significant reduction in blood pressure warrant specific programmatic attention for these occupational groups [14].

A major strength of this study is the high participation rate (87%) and use of real-world institutional data from a mandatory hospital-wide program, enhancing internal validity and relevance to similar healthcare settings.

However, several limitations must be acknowledged. First, the absence of a comparator group precludes causal inference. Second, individual adherence to exercise and educational components could not be measured. Third, random blood glucose was used instead of fasting glucose or HbA1c, limiting interpretation of glycemic trends. Fourth, the 6-month follow-up period may be insufficient to assess long-term sustainability of observed changes. Lastly, although occupational groups were analyzed separately, formal statistical interaction testing between time and job category was not conducted, limiting inference regarding differential intervention effects across professional roles.

In this retrospective cohort study, participation in a mandatory institutional health screening and exercise program was associated with favorable temporal trends in BMI, waist circumference, and cholesterol levels among hospital staff. Although statistically significant improvements were observed in BMI distribution, the absolute magnitude of change was modest, underscoring the need for sustained and longer-term interventions to achieve clinically meaningful impact. Variability across occupational groups and the absence of improvement in certain metabolic indicators highlight the need for tailored workplace health strategies. These findings should be interpreted as descriptive and hypothesis-generating rather than causal.

## Ethical statements

This study was performed under the ethical approval from Cicendo Eye Hospital Ethical Committee. All participants consented before all data were obtained.

## Funding

This research did not receive any specific grant from funding agencies in the public, commercial, or not-for-profit sectors.

## Declaration of competing interest

The authors declare that there was no conflict of interest.
